# Supplementary material for: Understandings of ultra-processed foods among adults with responsibility for household food activities in the United Kingdom: a qualitative study
Source: BMC Glob Public Health. 2026 Apr 16;4:35. doi: 10.1186/s44263-026-00263-0 (PMC13085418; doi:10.1186/s44263-026-00263-0)
Supplement: Supplementary file 4 — Supplementary Material 4. Codebook. [file 44263_2026_263_MOESM4_ESM.docx]

Codebook for UPF Interviews

| Name | Description |
| --- | --- |
| Definition of UPFs | Interviewee attempts to define UPFs, or to explain how they recognise them. |
| Additives and other ingredients | Discussion of UPF ingredients including additives, preservatives, emulsifiers, and other industrial formulations. |
| Difficult definition | The definition or the criteria for what makes a food UPF is difficult to understand. This could also be a clear example of confusion regarding the definition, even if the interviewee does not claim that the definition is difficult. |
| Examples of UPFs | When trying to define UPFs, these examples are given. |
| level of processing | Some people express there should be a scale of "how ultra processed" suggesting they see the category as heterogeneous. |
| never heard or think about it | they have either not heard about UPFs or don't really think about the term. it is at least not a common term they try to understand and interact with. |
| Not natural | UPFs are industrial products that do not come from natural food sources. |
| Preservatives and shelf life | Interviewees mention that UPFs have a lot of preservatives added to extend their shelf life. this is also sometimes a way to identify UPFs: when they have a long best by date, which tells the shopper they must have preservatives added. |
| Processed vs ultra-processed | Any discussion of the confusion or boundaries between the terms processed and UPF. |
| Scientific | The UPF category and concept is very scientific and perhaps overly scientific for the general population understanding. |
| Single ingredient | A simple term for minimally processed foods is single ingredient foods. this term seems to be common in social media. |
| Eating habits | Typically discussing whether they do/don't cook at home and how that affects UPF consumption. |
| how much UPF consumed | How much food the person claims is UPF in their diet. |
| I cook a lot or cooking is a good thing | Interviewee reports cooking frequently and/or views cooking as positive (e.g., enjoyable, healthier, important). |
| I don't cook a lot or cooking is too difficult | Interviewee reports cooking infrequently or describes cooking as difficult due to time, skills, motivation, or other barriers. |
| UPFs are a treat | Interviewee describes UPFs as an occasional treat/indulgence rather than a staple. |
| Food companies | Comments about food manufacturers/retailers and their role in producing, promoting, or shaping availability of UPFs. |
| They make UPFs for profit | Interviewee attributes UPF production/marketing to profit motives (e.g., cost-cutting, addictiveness, shareholder incentives). |
| Food culture and environment | Typically comes up with people who immigrated from other countries: how their food culture and supermarkets differ from the UK. There may be other observations about the difference between countries. A common example is that everything in the UK is packaged compared to other countries that have open air markets. |
| Everything in UK is packaged | Interviewee notes that foods in the UK are highly packaged compared with other contexts. Typically relates to someone who immigrated to the UK. |
| Other countries have healthier choices | Interviewee compares the UK unfavourably to other countries, suggesting healthier/more fresh options are more available elsewhere. May also relate to fewer fast food options in other countries. |
| People don't care | Expressions that the general public do not care about eating healthy, or health risks of UPFs. They prefer to stick to their typical habits and would not be interested to know whether UPFs are bad for them. |
| People eat this way because of how they're raised | Interviewee links dietary habits (including UPF consumption) to upbringing, family norms, or learned behaviour. |
| Govt Policy or Solutions | Discussion of government action/policies/solutions to reduce UPF consumption or improve diets. |
| Education policies | Suggestions for education-based interventions (e.g., public campaigns, school education, guidance on identifying/avoiding UPFs). |
| Fiscal policies | Suggestions for price-based policies (e.g., taxes, subsidies, pricing incentives) related to UPFs or healthier foods. |
| Marketing restrictions | Suggestions to restrict advertising/marketing/promotion of UPFs (e.g., to children, online influencers). |
| Oppose or Unsure of Policies to reduce UPFs | Interviewee expresses opposition to, uncertainty about, or ambivalence toward policies aimed at reducing UPF consumption. |
| Shop local | One response to the ubiquitous UPFs in all supermarkets is to shop local and get fresher foods that way. |
| Support Policies to reduce UPFs | Interviewee expresses support for policy measures to reduce UPF availability/marketing/consumption. |
| Habit changes | whether they have or haven't changed their UPF consumption recently, and potentially due to hearing more about UPFs. |
| need other healthy habits to balance | interviewee might say that people need to balance out their UPF consumption with other habit changes like exercise to balance out the calories and reduce risk of weight gain. |
| Health Issues | Any mention of health issues including how UPF consumption may cause or interact with health issues. |
| Don't know how UPFs affect health | It is either unknown to the individual or they believe the science is not settled on whether UPFs are bad for health. |
| Some UPF consumption should be ok | Interviewee expresses a moderation view: some UPFs are acceptable within an overall balanced diet. |
| UPFs bad for health | Interviewee states that UPFs are harmful to health (general or specific harms). |
| UPFs are addictive | Interviewee describes UPFs as addictive/compulsive or engineered to encourage overconsumption. |
| UPFs contribute to obesity | Interviewee links UPFs to weight gain/obesity or caloric overconsumption. |
| UPFs exacerbate health issues | Interviewee links UPFs to worsening existing health conditions (e.g., diabetes, gut issues, mental health). |
| UPFs neutral or beneficial to health | UPFs can be neutral or beneficial (e.g., fortification, convenience enabling intake, accessibility). |
| Intention to change UPF consumption | Statements about willingness or plans to change UPF purchasing/consumption. |
| Want to increase or keep same UPF consumption | Interviewee indicates no intention to reduce UPFs, or intends to maintain/increase intake. |
| Want to reduce UPF consumption | Interviewee expresses intention to reduce UPF purchasing/consumption (reasons, strategies, motivations). |
| requires discipline | Avoiding UPFs or reducing their consumption takes effort and/or discipline. |
| Key quotes | Quotes that stand out as strongly illustrating the speaker's point or capturing a general point of view about a topic in a way that is clearly and/or powerfully articulated. |
| Marketing | Mentions UPF marketing. |
| Reasons to eat UPFs | Mentions of reasons/motivations for consuming UPFs (e.g., time, cost, taste, access). |
| No good reasons to eat UPFs | Claims there are no acceptable reasons/justifications for eating UPFs. |
| There are good reasons to eat UPFs | Acknowledges acceptable reasons for eating UPFs (e.g., cost, time, access, taste, family needs). |
| Shopping priorities | Discussion of what interviewees prioritise when shopping for food (e.g., price, health, taste, convenience, labels). |
| Convenience or time | UPFs are helpful or a necessity because they provide convenience in a busy lifestyle. |
| hard to know what's healthy | Mentions it is hard to navigate the food environment and to know what is and isn't healthy. |
| Look for fresh or natural | Interviewee says that one of the priorities when they shop for food is to select food that is fresh and/or natural. minimally processed. may also include preferences for local fresh items rather than items transported a long distance. |
| Look for Healthy | Mentions thinking about healthy foods when they shop. |
| Look for tasty | Good tasting food is a key shopping priority. |
| Prefer higher end products | People will say they prefer higher end brands like Waitrose and M&S and stay away from Tesco and other budget markets. |
| Price | Discussion about cost of UPFs or non UPFs. interviewee may say they shop seeking value for money. any commentary about cost of UPFs. |
| Read labels for ingredients | Mentions reading ingredient lists (and/or nutrition labels) as part of decision-making. |
| Reading labels is difficult | Mentions difficulty interpreting labels (e.g., complex ingredients, unclear terms, time burden). |
| Reading labels is easy | Mentions confidence/ease in interpreting labels and using them in shopping decisions. |
| shop for variety | Try to vary the types of foods they buy and make as many different options as they can with what they buy. |
| Sources of info | Anything related to where interviewee has heard about UPFs. This could be media, mainstream news, online, social media, etc. |
| employer or uni | References their organisation discussed UPFs. |
| Friends & Family | References what other friends think about UPFs. |
| Mainstream news source | References mainstream news (TV/news sites/newspapers) as a source about UPFs. |
| Medical, health professional, or uni source | References medical, health professional, or university source. |
| Podcast source | References podcasts as a source of information about UPFs/diet. |
| Public conversation about UPFs | Whether public conversation about UPFs has increased or decreased in recent times. Are they hearing more about it these days. |
| Questionable or not trustworthy source | Describes some sources as unreliable/misleading (e.g., influencers, industry messaging). |
| Social Media source | References social media platforms as a source of information or exposure about UPFs. |
| Trustworthy source | Describes certain sources as credible/trustworthy (why/how). |
| TV Programmes and books | References TV programmes, documentaries, or books as information sources. |
| YouTube source | References YouTube as an information source about UPFs/diet. |
| Special diets | Mention of special dietary restrictions including vegan, keto, low FODMAP. |
| UPF taste | Mention of how UPFs taste and how taste influences consumption. |
| minimally processed tastes better | Could also include cooked from scratch, home cooked vs ready to eat. |
| UPF unsatisfying | There's something about eating UPFs that leave people feeling unsatisfied. whether that is energy levels or getting a brief sugar rush. They tend not to leave the consumer fully satisfied like a less processed meal does. |
| UPFs taste better | UPFs taste better or are more appealing than minimally processed options. |
| UPFs are a treat | UPFs are a tasty treat that might help make you feel better, if only in the moment. |
